# Bridging the Gap: A Roadmap to Breaking the Biological Design Barrier

**DOI:** 10.3389/fbioe.2014.00087

**Published:** 2015-01-20

**Authors:** Jacob Beal

**Affiliations:** ^1^Raytheon BBN Technologies, Cambridge, MA, USA

**Keywords:** synthetic biology, organism engineering, design, prediction, automation, metrology, calibrated flow cytometry

## Abstract

This paper presents an analysis of an emerging bottleneck in organism engineering, and paths by which it may be overcome. Recent years have seen the development of a profusion of synthetic biology tools, largely falling into two categories: high-level “design” tools aimed at mapping from organism specifications to nucleic acid sequences implementing those specifications, and low-level “build and test” tools aimed at faster, cheaper, and more reliable fabrication of those sequences and assays of their behavior in engineered biological organisms. Between the two families, however, there is a major gap: we still largely lack the predictive models and component characterization data required to effectively determine which of the many possible candidate sequences considered in the design phase are the most likely to produce useful results when built and tested. As low-level tools continue to mature, the bottleneck in biological systems engineering is shifting to be dominated by design, making this gap a critical barrier to progress. Considering how to address this gap, we find that widespread adoption of readily available analytic and assay methods is likely to lead to rapid improvement in available predictive models and component characterization models, as evidenced by a number of recent results. Such an enabling development is, in turn, likely to allow high-level tools to break the design barrier and support rapid development of transformative biological applications.

## 1 Introduction

The ongoing revolution in synthetic biology is bringing about a fundamental transformation in our relationship with the world of living organisms. One of the drivers of this revolution is the exponential rate of improvement in our ability to sequence, synthesize, and deliver nucleic acid sequences (Carlson, 2011). Improvements in reading and writing nucleic acid sequences in turn enable increasingly rapid modification of an ever-broadening set of organisms using a growing toolkit of biological mechanisms. Another key driver is the ongoing adaptation of engineering concepts originating in computer science and electrical engineering (e.g., Knight and Sussman, 1998; Hasty et al., 2002; Knight, 2003; Ferber, 2004; Canton et al., 2008). In particular, methods for generating and exploiting abstraction and modularity have enabled a “component-based” approach to engineering biological organisms that greatly simplifies the isolation and dissemination of useful biological mechanisms.

Viewed through the lens of a “design-build-test” cycle of iterative engineering, the first set of advances addresses build and test, while the second set addresses design. In both areas, progress is rapid, and advances are being encapsulated into tools that allow these improved methods to be widely applied. Between these two families of tools, however, there is a critical gap of growing importance: design, as it is currently typically practiced, is simply too imprecise. As has been widely and uncomfortably observed (e.g., Kwok, 2010), the behavior of engineered biological systems is frequently far different from predictions, typically due to some combination of unavailable information, inaccurate or incomplete models, and insufficiencies in available components. As a result, current practices for engineering biological systems typically require many iterations, and both time and cost increase rapidly with the complexity of the system to be engineered.

A number of recent results, however, indicate that these problems are becoming tractable to address. The goal of this manuscript is thus to analyze the problem landscape from a systems engineering perspective, producing a roadmap for breaking the biological design barrier and enabling the rapid and effective engineering of complex biological systems. Section 2 begins by analyzing the design-build-test cycle of biological engineering, developing an information-based metric for analyzing both the complexity of biological engineering problems and the efficacy of various engineering methodologies. Section 3 then applies this metric to analyze the relative potential impact from three complementary lines of tool development: high-throughput assays, improved device families, and predictive modeling and design. Narrowing the focus to predictive modeling, Section 4 examines the requirements necessary for an assay to effectively support predictive modeling, and illustrates how these can be satisfied through the example of a recently developed method for calibrated flow cytometry. Section 5 then connects assay capabilities back to the engineering of biological organisms by showing how calibrated flow cytometry has been used for intra-sample validation, to develop high-precision predictive models, and to support development of improved repressor devices. Finally, Section 6 summarizes and presents a roadmap for extending these capabilities to be readily applicable to a broad class of systems and organisms.

## 2 Quantifying Bottlenecks in Engineering Biological Organisms

**Figure 1:**
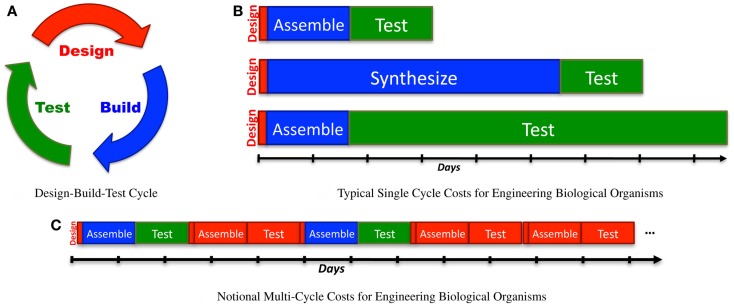
When analyzing synthetic biology against a classic design-build-test cycle **(A)**, analysis of a single cycle hides the cost of design, since the relative effort expended in a single cycle is typically quite low: **(B)** illustrates this with typical examples of time expended in a single cycle: a few minutes or hours of design, followed by construction of a design via BioBrick assembly (Knight, 2003) from existing sequence fragments or via an expedited order from a next-generation synthesis company, and then a 1-day test (e.g., a simple bacterial circuit) or a 1-week test (e.g., testing a memory circuit). A better measure, however, also takes into account the number of cycles required due to imprecision in design: **(C)** illustrates a sequence of design-build-test cycles in which any cycle that turns out not to be productive is accounted as a cost of problems in design.

Engineering is often conceived of as an iterative “design-build-test” process (Figure 1A). Although more mature engineering disciplines typically develop more sophisticated workflows [e.g., continuous integration (Duvall, 2007) and Agile processes (Larman, 2004) in software design, design for test (Crouch, 1999) in electronics, waterfall processes (INCOSE, 2010) for complex electromechanical systems], the classic iterative model is a good starting point for analyzing current synthetic biology approaches to the engineering of biological systems. Taking this approach, we can apply Amdahl’s law (explained in Section 1) to quantify process bottlenecks in the engineering of biological organisms. Here, the true cost of design becomes clear when it is considered as a multi-iteration search process, and can be estimated using the ratio of information required for a design to the information gained per test. This analysis can then be applied to assess the current ecosystem of design tools, identifying critical gaps and opportunities.

**Figure 2:**
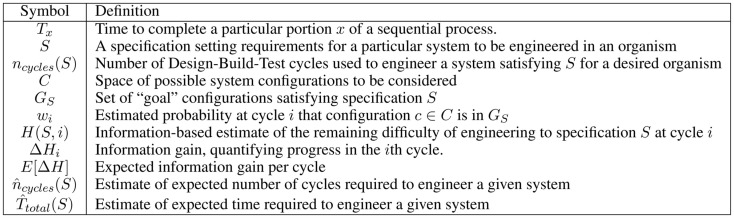
**Table of significant notation used for analysis of organism engineering bottlenecks**.

For the convenience of the reader, Figure 2 provides a table of notation used in this section.

### 2.1 Quantifying bottlenecks: Amdahl’s law

In computer science, Amdahl’s law (Amdahl, 1967) is frequently used for analyzing cost–benefit tradeoffs in optimizing the speed of complex processes. In essence, Amdahl’s law is simple arithmetic: if a particular stage of a sequential process takes time *T_stage_* to complete, and the remainder takes a total time *T_total_* − *T_stage_*, then optimizing that stage to be *k* times faster gives an overall speedup of:

(1) $$k_{\mathrm{total}}=\frac{T_{\mathrm{total}}}{\left(T_{\mathrm{total}}-T_{\mathrm{stage}}\right)+\frac{T_{stage}}{k}}$$

In other words, the efficacy of improving one stage in a process (e.g., by increasing parallel throughput) is bounded by the fraction of time spent in the other stages.

For example, if there are two stages, the first taking 2 days and the second taking 1 day, then a *k* = 2 speedup of the first stage, to 1 day, will improve the total speed by 3/1 + 1 = 1.5 times, while a *k* = 2 speedup of the second stage to half a day will only improve the total speed by 3/2 + 0.5 = 1.2 times.

Let us consider how this analysis applies to the design-build-test cycle (Figure 1A) for one of the most common process workflows in the practice of synthetic biology:

- *Design* proposes a set of nucleotide sequences that will be assayed with the aim of advancing toward some engineering goal.
- *Build* physically realizes the desired nucleotide sequences through some combination of protocols such as for synthesis, assembly, editing, and purification.
- *Test* assays the results of gene expression from these nucleotide sequences under various experimental conditions.

This general schema covers much of the practice of synthetic biology, from developing sensors to tuning chemical synthesis, from directed evolution to circuit engineering, from microbes to specialty mice.

A simplistic analysis could simply consider the time required for each of these stages in a cycle:

(2) $$T_{\mathrm{cycle}}=T_{\mathrm{design}}+T_{\mathrm{build}}+T_{\mathrm{test}}$$

Applying Amdahl’s law to this formula, we can determine where there is the most opportunity for improvement in a given engineering cycle. At present, this is typically dominated by building or testing, which frequently require days to weeks, depending on the particular constructs and organisms involved, as illustrated in Figure 1B. From the single-cycle perspective, then, design would appear to be only a niche concern, relevant only for subareas with particularly high computational requirements, such as rational design of proteins.

Since there may be many cycles, however, a better approximation of the time required for engineering a biological organism to meet some particular specification *S* is:

(3) $$T_{\mathrm{total}}\left(S\right)=n_{\mathrm{cycles}}\left(S\right)\times T_{\mathrm{cycle}}$$

The number of cycles, in turn, is affected by the *quality* of choices made during each design phase. For example, design choices that lead to dead ends or simply turn out to fail may result in unproductive cycles that may reasonably have their costs assigned to design, as illustrated in Figure 1C.

Since every engineering project is likely to face different challenges, how can we analyze the effect of design methods on the number of cycles? At a fine grain, of course, we cannot predict which cycles will be unproductive – otherwise, they would not happen in the first place. We can, however, quantify the efficacy of any engineering method by viewing the sequence of design-built-test cycles as an incremental search through the space of possible designs. Viewed in this way, the *expected* performance of an engineering method can be analyzed using various well-established methods (Russell and Norvig, 2003) from artificial intelligence and information theory.

### 2.2 Information-based estimation of engineering cycles

Let us consider the engineering process as a search through the space of possible designs. This design space *C* consists of the set of all possible system configurations within the scope of consideration. For any given design specification *S*, the subset *G_S_* ⊆ *C* is the set of “goal” configurations that sufficiently satisfy the specification (we will assume that these are simple to recognize when tested). The search process is thus an attempt to either identify at least one member of *G_S_* or to determine that the set is empty.

For example, consider engineering of a metabolic pathway that expresses five enzymes: if each enzyme’s expression is driven by a constitutive promoter and 5′UTR chosen from rationally engineered libraries of 10 of each type (e.g., via Salis et al., 2009), and these functional units are joined to form a single plasmid, then there are 10^5^ × 10^5^ × 5! × 2^5^ = 3.8 × 10^13^ possible configurations (10^5^ for five independent choices from a set of 10 promoters, times 10^5^ for five choices from 10 5′UTRs, times five-factorial possible orderings, times 2^5^ for five independent choices of plus or minus strand). For another example, consider engineering as a circuit of 7 repressors drawn from the orthogonal library of 20 in Stanton et al. (2014). Selecting the repressors and organizing their functional units on a plasmid gives $\left(\begin{array}{c} 20\\ 7 \end{array}\right)\times 7!\times 2^{7}=5.0\times 10^{10}$ possible configurations [there are $\left(\begin{array}{c} 20\\ 7 \end{array}\right)$ possible combinations of library repressors, times seven-factorial possible orderings, times 2^7^ for seven independent choices of plus or minus strand].

Prior knowledge about the likelihood of configurations being in *G_S_* can be modeled by a normalized weight function *w*_0_: *C* →[0, 1], such that configurations known not to be in *G_S_* map to 0 and those most likely to be in *G_S_* map to 1. After each cycle, this function is updated to a new *w_i_* based on the information learned from that cycle’s tests. Any cyclic engineering process may then be modeled by the following meta-algorithm:

1. Select a set of candidate configurations *c* ⊆ *C*, on the basis of *w_i_*.
2. Build and test all members of *c*.
3. If some *c* ∈ *G_S_*, then *SUCCEED*.
4. Incorporate knowledge gained from the tests to generate *w_i_*_+1_.
5. If *w_i_*_+1_ is uniformly zero, then *FAIL*, since *G_S_* has been demonstrated to be empty. Otherwise, return to step 1.

Using information theory, we can quantify how hard it is to find goal configurations by considering the selection of candidates at random^1^. By the standard definition of entropy, the number of bits *H*(*S*, *i*) for the *i*th cycle of design toward some specification *S* is thus:

(4) $$H\left(S,i\right)=\log_{2}\left(\frac{\sum_{c\in C}w_{i}\left(c\right)}{\sum_{c\in G_{S}}w_{i}\left(c\right)}\right)$$

where *w_i_*(*c*) is the estimated likelihood of configuration *c* belonging to *G_S_* given the information available at cycle *i*. This is thus an *information-based* measure of the progress of engineering a system over time.

Note that better information about the likelihoods of configurations belonging to *c* reduces the number of bits, until in the limit *w_i_* puts a non-zero weight only on members of *G_S_*. At this point, the number of bits is zero and success is certain. Complementarily, lack of information and sparse goals increase the number of bits toward an upper limit of log_2_|*C*|. Thus, the metabolic pathway example above is an engineering problem of up to 45.1 bits and the circuit example up to 35.5 bits.

The efficacy of an engineering method may then be evaluated in terms of the number of bits of information obtained per cycle, formally:

(5) $$\Delta H_{i}=H\left(S,i\right)-H\left(S,i+1\right)$$

In general, the number of bits remaining should decrease with each additional assay, giving a positive Δ*H_i_*, and the greater the decrease in entropy, the better the efficacy of the method.

To illustrate these notions of information gain, consider the metabolic pathway example, beginning with no information about the appropriate expression levels. Some examples of information gain:

- Determining that enzyme #2’s 5′UTR should be in the upper half of the expression range gains 1 bit of information.
- Determining which promoter should be used for enzyme #2 gains 3.32 bits of information.
- Determining that enzymes #3 and #4 should be expressed with matching promoter/5′UTR combinations gains 6.64 bits of information.
- Using insulators that eliminate the effect of ordering and strand choice gain 11.91 bits of information.
- High-throughput screening of 10^10^ arbitrarily chosen combinations gains only 0.0004 bits of information.

Notice that in these examples, the model-driven information gains are much greater than the gains from brute-force screening – even with a rather large throughput. The relative balance of model-driven and exploration-driven approaches depends on the scale of the problems. For example, if the pathway contained only three enzymes rather than five, it would only be a 25.5-bit configuration space and could be screened completely using less than 10^8^ combinations. Notice also, that some of these information gains are “one shot” while others are not. For example, using insulators is a single choice, and cannot narrow the design space further. A method that can incrementally refine expression level choices, however, might be applied iteratively to complete the entire design, providing a consistent expected information gain *E*[Δ*H*] per cycle.

A conservative estimate of the expected number of cycles ${\hat{n}}_{cycles}(S)$ required to engineer specification *S* by a particular engineering method with an expected information gain of *E*[Δ*H*] per cycle may thus be computed by assuming there is only a single possible solution in *G_S_*. Under this assumption, the estimated number of cycles is:

(6) $${\hat{n}}_{\mathrm{cycles}}\left(S\right)=\frac{H\left(S,0\right)}{E\left[\Delta H\right]}$$

Returning to the original equation, we may thus estimate the expected time to be required to engineer a biological system to satisfy a given specification *S* as:

(7) $${\hat{T}}_{\mathrm{total}}\left(S\right)=\frac{H\left(S,0\right)}{E\left[\Delta H\right]}\times T_{\mathrm{cycle}}$$

## 3 Potential Impact of Improved Engineering Tools

Let us now bring this analysis back to the original question: what are the bottlenecks in engineering biological organisms, and the key points for investigation to improve the situation? Having cast the problem of engineering biological organisms in information-based terms, we can see that there are only three terms in the equation for the estimated time for engineering. Each term then implies a particular strategy for improving speed:

1. decreasing the amortized time per configuration assay by decreasing amortized *T_cycle_* (e.g., by decreasing the time per cycle or by running more cycles in parallel),
2. decreasing the effective bits *H*(*S*, 0) required by vastly enriching the number of acceptable “goal” configurations, and,
3. increasing the number of bits *E*[Δ*H*] of design-constraining information expected to be gained per configuration assay.

The first strategy focuses on the “low-level” tools aimed primarily at the build and test aspects of the engineering cycle. Capabilities in this area are increasing rapidly, but there are sharp limits in what can be enabled by this strategy alone. The second and third strategies relate more to “high-level” design tools for mapping from a specification to a candidate configuration in *C*. Here, there is a critical gap stemming from the difficulty of predicting the behavior of a configuration, which we analyze along with emerging opportunities for bridging this gap.

### 3.1 Decreasing time per assay: High-throughput screening

The time required for a cycle of testing is strongly limited by the underlying physical processes involved in build and test. Even if fabrication time might be greatly reduced, the time for an *in vivo* assay is an immutable bottleneck set by the inherent dynamics of the organism being assayed. This can potentially be mitigated through *in vitro* assays, assuming that there are models and design tools that can mitigate the effect of differences between *in vitro* and *in vivo* environments. However, even the fastest *in vitro* assays (e.g., Carlson et al., 2012; Sun et al., 2013), which operate on a time scale of only hours, are physically limited by the dynamics of the systems to be assayed. Thus, serial improvements to throughput are likely limited to around one order of magnitude increase in throughput relative to typical cell culture assays.

For the complementary approach, decreasing the amortized time per configuration assay through massive parallelization of high-throughput screening, typical practice is much farther from physical limits, giving the opportunity for much more improvement in throughput. High-throughput parallel screening is already a subject of much investigation, a recent review of which may be found in Dietrich et al. (2010). Significant research and commercial development is being invested in multiple tools designed to increase both capabilities and accessibility, including in robotics (e.g., Vasilev et al., 2011; Hillson et al., 2012; Linshiz et al., 2012), microfluidics (e.g., Kong et al., 2007; Gulati et al., 2009), evolutionary methods (e.g., Esvelt et al., 2011; Cobb et al., 2012; Lynch and Gill, 2012), and languages for low-level specification and data exchange (e.g., Bilitchenko et al., 2011; Galdzicki et al., 2012; Myers, 2013). This portion of the tool ecosystem is thus quite healthy and rapidly evolving.

**Figure 3:**
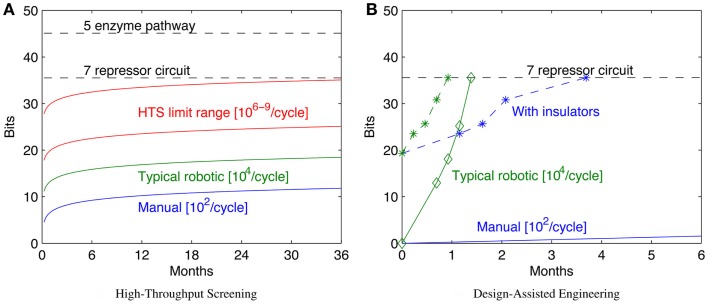
**Decreased time per assay has sharply limited benefits, as illustrated by comparison of the bit size of example moderate- complexity circuits to rates of configuration space exploration (A)**. Solid lines show the number of bits of configuration space that can be assayed in a sequence of 1-week cycles with various methods (starting with a single parallel assay at week 1), while dashed lines show the complexity of the example circuits in Section 2. Improving models and components can dramatically reduce the required number of assays: **(B)** illustrates how a model-driven design process for the seven-repressor circuit might progress incrementally by breaking the system into three sequentially engineered subsystems (solid lines with diamonds marking sequence steps; manual is blue, robotic is green), and how that might be further improved with insulators that eliminate the effect of ordering and strand choice (dashed lines with stars marking sequence steps; manual is blue, robotic is green). For **(B)**, progress toward completion is shown by graphing *H*(*S*, 0) − *H*(*S*, *i*) for each circuit. Note that for the lowest manual line, no diamond appears because the first step is not completed for more than 6 months.

Increasing throughput, however, has sharp limits in efficacy, because the configuration space grows exponentially with the number of bits, which in turn typically scales linearly with the complexity of the system being engineered. As a result, “brute force” approaches through high-throughput screening can readily solve problems up to a certain number of bits, but are effectively useless when addressing problems only a little bit larger. Figure 3A illustrates this scaling problem by comparing the circuit and metabolic pathway examples from the previous section with the size of configuration space that can be explored with a 1-week build/test cycle at various rates of high-throughput screening. When samples are prepared manually, the rates that can be effectively sustained for a single laboratory worker are on the order of 10^2^ configurations per cycle (e.g., a few replicates in 96-well plates). A well-pipelined fluid-handling robotics cell can prepare such assays continuously, raising the rate to around 10^4^ configurations per cycle. Other techniques can potentially raise the rate by orders of magnitude, but there are limits due to various pragmatic barriers: Dietrich et al. (2010) calculated effective parallelism limits of high-throughput screening to be around 10^6^–10^9^ configurations per screening assay. As can be seen, even at high rates of throughput, only moderately complex configuration spaces can be explored in a reasonable period of time.

High-throughput screening is still a valuable component of the toolkit for engineering biological organisms. As the capacity and accessibility of high-throughput screening continue to increase, brute-force screening is likely to be sufficient for realizing a large number of “low hanging fruit” applications, particularly certain classes of medical, sensor, and chemical synthesis applications where the engineered organism only needs to operate for a relatively short period of time in a tightly controlled and isolated environment.

When contemplating longer lived systems in less controlled environments, however, it is reasonable to expect that there will generally be a need for more complex mechanisms that can ensure safety, stability, and effective operation under a range of conditions. Evolutionary methods are often proposed as a means of obtaining continuous incremental improvement toward such more complex systems (e.g., Cobb et al., 2012; Lynch and Gill, 2012). Great progress has been made with these methods, and they have proven highly effective for solving problems with simple specifications in permissive spaces (e.g., Chudakov et al., 2010; Brustad and Arnold, 2011). Computer science researchers, however, have identified a key set of open problems that must be addressed in order for evolutionary methods to be effective for complex engineered systems of any sort, biological, or otherwise (Forrest and Mitchell, 1993; O’Neill et al., 2010). It is not yet clear whether it is even possible to solve these problems for synthetic biology, and unless they can be solved, effective engineering of even moderately complicated synthetic biology systems will necessarily require methods that include a more model-driven approach to design.

### 3.2 Decreasing required assays: Improving components and models

The other half of Equation (7), estimating number of cycles, addresses how large the effective configuration space is and how effectively an engineer can apply assays in searching for a goal configuration. Here, there is a major and well-recognized gap in the current tool ecosystem: given the current set of available parts and models, it has not generally been possible to accurately predict the behavior of multi-element designs except in certain special cases (Kwok, 2010; Lux et al., 2012).

At higher levels of abstraction, mapping from behavior specifications for cell aggregates or individual cells to specifications of candidate regulatory networks intended to implement those specifications, there are a number of candidate tools and approaches. These encompass a wide variety of models, addressing computation and control, metabolic synthesis, and even the development of structure and patterns (e.g., Pharkya et al., 2004; Beal and Bachrach, 2008; Pedersen and Phillips, 2009; Moriya et al., 2010; Beal et al., 2011; Marchisio and Stelling, 2011; Yousofshahi et al., 2011; Huynh et al., 2013). Despite some notable current gaps^2^, this portion of the design space appears to be generally susceptible to a variety of methods from computer science, electronic design automation, and signal processing.

Because of the difficulty in predicting the behavior of multi-element designs, however, no high-level design tool is currently capable of supporting an effective search of a large configuration space. Instead, at present, these tools generally either stop at the design of an “abstract” circuit that can be realized into many different configurations (e.g., Beal et al., 2011), require collections of devices, and/or data about devices that are not currently available (e.g., Pedersen and Phillips, 2009; Rodrigo et al., 2011; Yaman et al., 2012; Huynh et al., 2013), or can generate large numbers of candidate configurations with no reliable means of distinguishing, which (if any) are likely to actually meet the specification (e.g., Czar et al., 2009; Bilitchenko et al., 2011). In all cases, however, the fundamental problem is a lack of precision in the available models for crossing the gap between a sequence specification and a prediction of the expression that will result from this sequence in context.

There are two basic approaches to addressing this problem, corresponding to the numerator and denominator of the estimated number of cycles. The first approach is to decrease the effective complexity of the configuration space *H*(*S*, 0) by some combination of:

1. increasing the signal-to-noise characteristics of intended component interactions, and
2. decreasing the effect of unintended interactions between components and their environment.

Improvements of either type decrease the degree of coupling between choices in a configuration, i.e., the likelihood of incompatibility between choices: the lower the likelihood of two independent design choices being contained within *G_S_*, the higher the degree of coupling between choices, because any given design choice must more carefully take into account the other choices that have been made. Coupling and effective complexity have a well-established relationship in both complexity theory (Kanefsky and Taylor, 1991; Hogg et al., 1996) and statistical physics (Krzakala and Kurchan, 2007; Dall’Asta et al., 2008; Zdeborová, 2008): as degree of coupling decreases, the structure of the configuration space undergoes a dramatic phase transition, such that it becomes easy to either find a goal configuration or determine that none exists. Put more intuitively: it is much easier to engineer with components that are less delicate.

In synthetic biology, a number of different ongoing efforts are aimed at improving signal-to-noise and at decreasing unintended interactions. Methods for improving signal-to-noise are currently largely focused on improving the number of available orthogonal high-amplification regulatory mechanisms. A number of approaches are being pursued, including recombinases (e.g., Bonnet et al., 2013), homolog mining (e.g., Stanton et al., 2014), and high-performance synthetic repressors (e.g., Kiani et al., 2014). At the same time, methods for decreasing unintended interactions are being investigated across a large range of targets, from decreasing promoter/5′UTR interaction (e.g., Lou et al., 2012; Mutalik et al., 2013), to making interactions between functional units more predictable by cotransfection (Beal et al., 2012, 2014), to construction of entirely orthogonal systems of transcriptional machinery (Neumann et al., 2010; Schmidt, 2010). It is unclear at present, however, how quickly these efforts can progress, how well their goals can be achieved, and in which classes of organisms.

The complementary approach to reducing *H*(*S*, 0) is increasing *E*[Δ*H*], so that the search for functional configurations can be better guided by improved models of components and their intended and unintended interactions. More precise predictive models can improve the rate of information gain in a number of ways, notably including:

- Entire subspaces of non-functional configurations can be eliminated from consideration without any assay.
- An assay of one configuration can provide information (adjustments to *w_i_*) about a large family of related configurations.
- Complicated systems can be decomposed hierarchically or thematically into subsystems whose details can be designed independently.

Figure 3B illustrates an example of how a model-driven engineering process might exploit such techniques, using the example of a seven-repressor circuit from Section 2. For this example, let us assume the same assay rates as for the high-throughput screening comparison in Figure 3A, but instead of a brute-force search of the space, the circuit is broken into three modular subsystems and each of these engineered sequentially. First, all possible implementations of a subsystem of three repressors are assayed, followed in turn by two more two-repressor subsystems, covering the circuit. Note that the information gain for each stage is not uniform, because of how the combinatorics of possible remaining options differs. The repressors are then assayed for signal levels and orthogonality, and this information used to pick the best three compatible candidates for each subsystem: all combinations of these candidates are constructed, and the best version accepted, assuming that it is sufficiently functional. Such a process may not identify the optimal system, but instead makes incremental progress toward identifying a “good enough” system, as long as the models are predictive enough and component coupling low enough to enable the subsystem assays to effectively constrain the candidates for the final design. The example design would still be too complex to search effectively with manual assays (an expected time of nearly 5 years at the specified rate), but can be readily tackled with fluid-handling robotics. Additional improvements to the design space might further improve the effective bits per assay: for example, with insulators that eliminate the effect of ordering and strand choice, even manual assay preparation is a viable strategy.

Construction of predictive models to enable such modular approaches to engineering has been a major goal of synthetic biology since its inception (e.g., Knight and Sussman, 1998; Elowitz and Leibler, 2000; Gardner et al., 2000; Weiss, 2001). Significant progress has been made in predictive engineering of behaviors of individual circuit components (e.g., Salis et al., 2009; Liu et al., 2012; Lou et al., 2012; Borujeni et al., 2013; Kosuri et al., 2013). Successful prediction results for the interaction of multiple components, however, have been rare and generally applicable only to special cases (e.g., Rosenfeld et al., 2007; Stricker et al., 2008; Ellis et al., 2009; Tabor et al., 2009).

In the next section, we will argue that a major barrier to progress in predictive modeling has been the unavailability of sufficient assay methods. Recent improvements in assay methods, however, have enabled previously unattainable precision in quantification. Improved precision then enables better predictive models, supporting new and more effective approaches to both the engineering of multi-component circuits (Beal et al., 2014; Davidsohn et al., 2014) and to the engineering of individual components (Kiani et al., 2014). All of this amounts to a significant increase in the amount of information that can be gained per assay, entirely complementary to high-throughput screening and component improvement, and offers the opportunity for rapid advancement in the speed of biological organism engineering.

## 4 Measurement Assays to Support Model-Driven Engineering

Let us now focus on the foundation for model-driven engineering: sufficiently powerful measurement assays. Effective quantitative modeling is impossible without being able to obtain accurate and precise measurements of the phenomena to be modeled. This section thus first analyzes what is required for an assay aimed to support model-driven engineering, then presents in detail an example of a recently developed method, calibrated flow cytometry, that satisfies these requirements.

### 4.1 Assay requirements for effective modeling

Any effective synthetic biology program of quantitative modeling requires assays with the following capability: *absolute unit measurements from large numbers of single cells*. To see why, let us break this statement up and consider it one point at a time.

#### 4.1.1 Absolute unit measurements

Much of the prior work in both systems and synthetic biology reports results in relative or arbitrary units – in other words, values that are not tied to any SI unit. This is an unusual practice for a scientific field, but so widespread that it goes virtually unremarked upon. When relative units are used, the focus of scientific reproducibility is not the individual measurements, but their relationship, e.g., the fold-repression exhibited by a transcription factor.

Relative units, however, cannot be combined across different experiments. This means that models of individual components or interaction phenomena cannot in general be combined to predict the behavior of new configurations. For quantitative models to be portable across experiments, systems, and laboratories, they must therefore be based on measurements tied to some absolute standard, preferably in SI units^3^.

#### 4.1.2 Single cells

**Figure 4:**
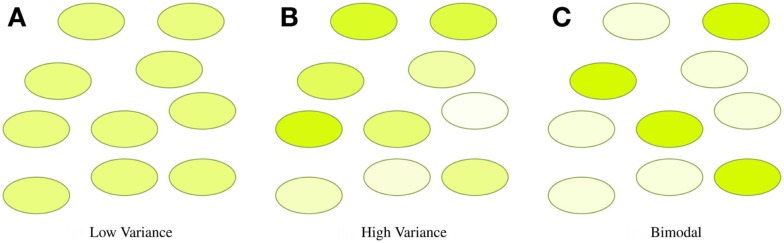
**Assays that measure population means or totals cannot distinguish between even radically different distributions of expression**. For example, the tight **(A)**, broad **(B)**, and bimodal **(C)** distributions illustrated above all have the same mean and total fluorescence.

In many synthetic biology systems, there is significant variation in the behavior of individual cells. Many assays, however, obtain only a cumulative or mean measurement across an entire population of cells. As a result, such population-level assays cannot distinguish between radically different distributions of values. For example, Figure 4 illustrates three very different distributions of fluorescence: a tight homogeneous distribution, a highly variable unimodal distribution, and a strongly bimodal distribution, all of which have the same mean and total fluorescence over the population. Since high cell-to-cell variation is so common, and has been shown to be important in understanding many systems (e.g., Rosenfeld et al., 2005; Beal et al., 2014), effective quantitative modeling requires assays that can obtain their absolute measurements from individual cells.

#### 4.1.3 Large numbers of cells

Finally, not only is it important to take measurements of individual cells, but to obtain them from *large* numbers of individual cells. The reason is that there are often multiple different phenomena driving different modes of variation in the behavior of a population of cells. Some of the key classes of phenomena driving variation include:

- Inherent process stochasticity: e.g., transcription, translation, replication.
- Cell-to-cell differences: e.g., size, cycle state, health, mutations, location.
- Protocol stochasticity: e.g., transfection variation, insertion site.
- Protocol execution issues: e.g., reagent variation, contamination, instrument drift.

In modeling and engineering a system, each of these classes must be handled differently. For example, inherent stochasticity has largely uncorrelated effects on individual genetic components, while cell-to-cell differences have highly correlated effects on all of the components within an individual cell. Likewise, protocol stochasticity can often produce distributions of individual cell behaviors predictably controlled by variables in the protocol, while protocol execution issues are more generally unpredictable and must be detected and appropriately compensated for.

**Figure 5:**
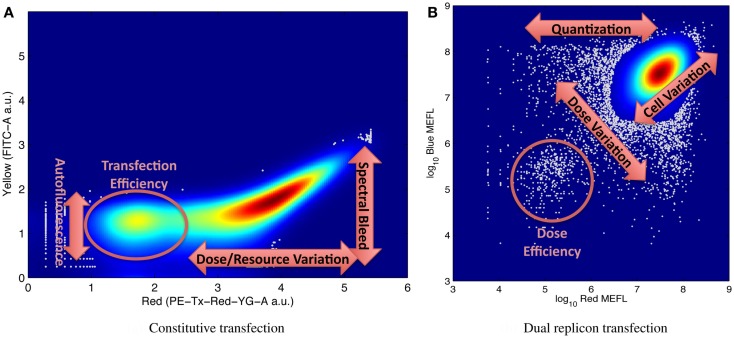
Examples of flow cytometry data showing complex population variation driven by multiple phenomena, with labels on key portions of the distribution used for estimating model parameters: **(A)** transient transfection of a single constitutively expressed fluorescent protein, from Adler et al. (2014), and **(B)** cotransfection of two replicons with constitutively expressed fluorescent proteins, from Beal et al. (2014). Both graphs indicate distribution density with color, with dark red indicating the maximum density, and outlier data (those in areas with less than 5% of maximum density) indicated by gray dots.

It is for this reason that large numbers of individual cell measurements are needed, in order to be able to accurately distinguish and resolve multiple modes of variation. Figure 5 shows illustrative examples of complex distributions of cell behaviors, labeled to indicate aspects of the distribution that can be used to quantify various significant mechanisms for understanding system behavior. For example, Figure 5A, from Adler et al. (2014) shows transient transfection of a plasmid constitutively expressing red mKate fluorescent protein into HEK293 mammalian cells. Fluorescence is measured on two channels of a flow cytometer, one configured to measure mKate, the other to measure the yellow EFYP fluorescent protein. The distribution is strongly bimodal, with non-transfected cells expressing little red and transfected cells strongly expressing red: the relative number of non-transfected cells is proportional to transfection efficiency, while the range of expression in the transfected population indicates the range of variation in dose and resources from cell to cell. At the same time, the second channel can be used to quantify both autofluorescence (from the non-transfected cells) and the degree of spectral overlap between yellow and red channels of the instrument in its current state (from the location of the inflection point in the transfected population). Similar mechanisms are at work in Figure 5B, taken from the data of Beal et al. (2014), showing a cotransfection of two Sindbis RNA replicons into BHK-21 mammalian cells, one constitutively expressing mKate, the other EBFP2. The relative size of the lower-left population of cells expressing minimal fluorescence is proportional to the transfection efficiency, and cell-to-cell variation in resources is proportional to the variance along the diagonal axis. In addition, the number of cells and variance of the off-axis component of the distribution indicate the size and distribution of the initial dose, since all doses are quantized (i.e., it is not possible to transfect a fractional replicon) and smaller initial doses have higher variance.

The exact number of observations required to quantify mechanism models from distributions such as these depends on the structure of the distributions involved. In general, however, more samples are required to obtain the same level of accuracy for more complex distributions or distributions with less clearly separated components. To give a sense of scale, the experiments reviewed in this section and the next range from around 30,000 to 1,000,000 samples per condition assayed, depending on the particular goals and requirements of the assay.

From these arguments and examples, we can see that an assay that can obtain absolute measurements from a large number of single cells has the potential to provide a great deal of insight into the behavior of a biological system. Moreover, it is likely to be difficult to make accurate predictive models of cell behavior without being able to use such a capability to separate and quantify the different modes of variation affecting cell behavior.

### 4.2 Calibrated flow cytometry

Until recently, there has been no readily accessible assay for gene expression that could satisfy the requirement for absolute measurements of large numbers (on the order of 10^5^) of single cells. This has changed, however, with the development of the TASBE method for calibrated flow cytometry (Beal et al., 2012), which builds on pre-existing calibration methods to enable high-throughput measurement of equivalent absolute units from multiple fluorescent species (often proteins, though other classes of molecule can also be used).

As an instrument, flow cytometers already fulfill two of the three assay requirements, since they break a sample into individual particles (many corresponding to individual cells) and take fluorescence measurements on multiple channels simultaneously from large numbers of those particles. Better yet, flow cytometers have become widely available, and many flow cytometers have high-throughput screening capabilities that make it easy to evaluate many samples in a short time. The measurements produced, however, are in arbitrary units, which can vary wildly depending on the machine and its settings and which are subject to drift over time. Thus, in order to transform flow cytometry into an assay capable of supporting modeling, it is necessary to add calibration controls that can enable a reliable mapping from relative to absolute units.

The TASBE method (Beal et al., 2012) builds on prior methods for flow cytometry calibration (Roederer, 2001, 2002; Schwartz et al., 2004; Hoffman et al., 2012), enhancing them to allow equivalent units to be read from all channels of a flow cytometer. The TASBE method is modular and can be incorporated as an extension to nearly any other flow cytometry protocol. Cells should be prepared and gated to remove non-cell particles as dictated by the base protocol; the TASBE method just requires that each experiment also include measurements from a set of calibration controls (some of which should already be part of any experiment).

**Figure 6:**
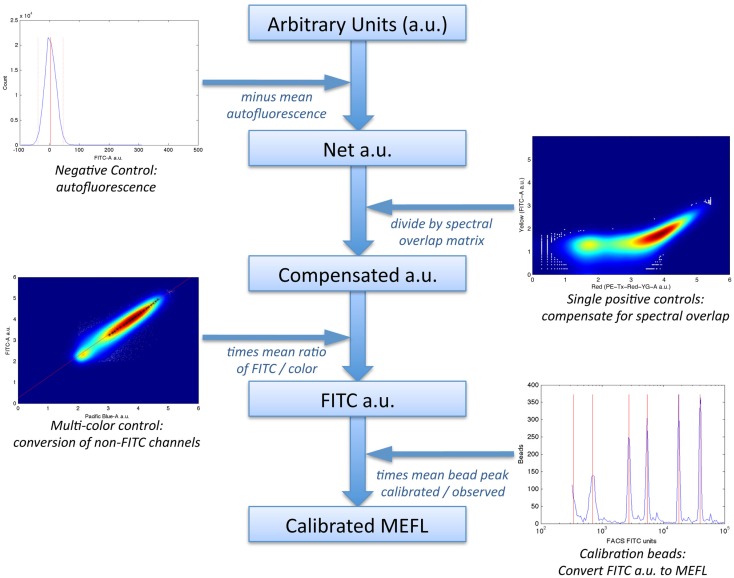
The TASBE method (Beal et al., 2012) for calibrated flow cytometry uses four controls: correction for autofluorescence and spectral overlap is computed from the negative and single-positive controls. Calibration beads provide a conversion from arbitrary units to molecules of equivalent fluorescein (MEFL) on the FITC channel, and multi-color controls allow all other channels to be converted to equivalent FITC units and thence to MEFL. Data shown are from sample material on Adler et al. (2014).

In particular, the method uses a set of four controls to compute a calibrated “color model” for converting data from arbitrary units to absolute units, as illustrated in Figure 6:

1. a negative control, to quantify autofluorescence;
2. single-positive controls for each fluorescent species, to quantify spectral overlap;
3. fluorescent beads calibrated to an absolute standard of Molecules of Equivalent Fluorescein (MEFL), such as SpheroTech RCP-30-5A beads (SpheroTech, 2001);
4. for each fluorescent species not measured in the FITC channel, a multi-color control with equivalent co-expression of that species and the species measured in the FITC channel.

The first two controls are used to remove fluorescence contamination from the measurements; the latter two controls are used to convert to absolute units.

#### 4.2.1 Compensation for fluorescence contamination

Fluorescence measurements are contaminated in two ways. First, cells (and the medium in which they are suspended) have some degree of autofluorescence, adding a consistent background to any fluorescence measurement. Second, there is often overlap in the excitation and emission spectra of fluorescent species, such that the measurements for each species will include “spectral bleed” proportional to each other species’ concentration and degree of overlap. The amount of overlap depends on particular fluorescent species and the configuration and settings of the flow cytometer.

**Figure 7:**
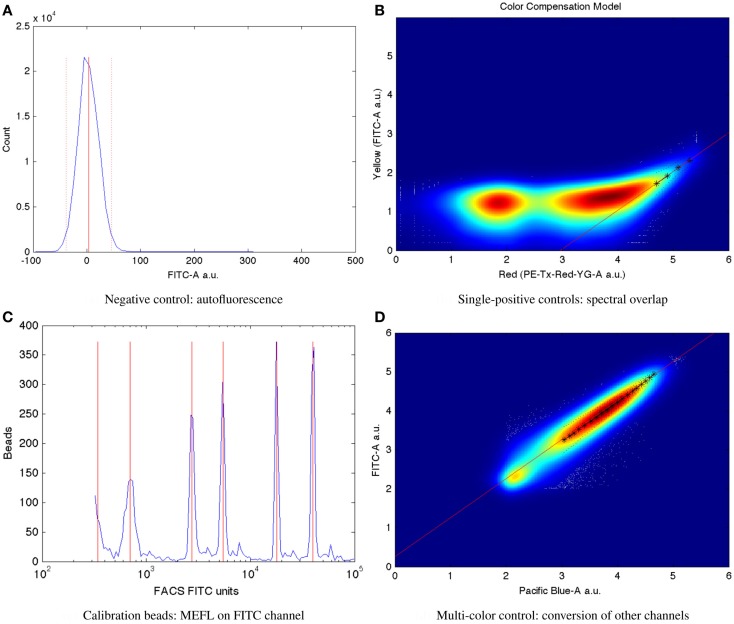
**Larger versions of the sample controls shown with the TASBE method workflow in Figure 6**. **(A)** Computation of autofluorescence from negative control. **(B)** Computation of spectral overlap from single positive control. **(C)** Computation of from MEFL conversion factor from calibration beads. **(D)** Computation of color conversion factor from multi-color control.

Mean autofluorescence on each channel can be estimated from a negative control, either wild-type or a null transfection/transformation (null is preferred over wild-type, because some cells’ fluorescence properties change in response to the stress of transfection/transformation protocols). Autofluorescence is typically normally distributed; Figure 7A shows a typical example of low autofluorescence (from untransfected HEK293 cells), computing both the mean (solid red line) and two standard deviations (dotted red lines).

Once autofluorescence has been quantified, spectral overlap can be estimated from strong constitutive expression of each fluorescent protein individually. With a single protein being expressed, any fluorescence observed significantly above autofluorescence in any other channel must be the result of spectral bleed. This is a linear effect, and thus may be estimated from the mean ratio of the two measurements for highly expressing particles after autofluorescence is removed. Figure 7B shows an example of computing the spectral bleed from strong constitutive expression of the red fluorescent protein mKate into the FITC channel (in this case intended to be used for quantifying EFYP expression), finding an approximate bleed of around 0.1%. Note that this is a relative measure, depending on the settings of both channels involved, rather than an absolute measure of the percentage of energy contaminating. This distinction is important because the purpose is to be able to use the (relative) measurement on one channel to correct the (relative) measurement on the other.

Once autofluorescence and spectral bleed have been quantified, they may be removed using an affine transform, as described in Roederer (2001, 2002). High spectral bleed, however, still results in increased noise, as described in Roederer (2002). For high-precision quantification, a best-practices standard for spectral bleed is thus <1%, though higher levels can be tolerated for some purposes. The writeup in Beal et al. (2012) also describes a method for selecting species/channel combinations with minimal overlap. With presently available instrumentation and fluorescent species, it is typically possible to meet this best-practices standard for two to four species, depending on the instrument and its configuration.

Finally, note that while some flow cytometers have features to perform their own spectral compensation, it is generally better to take data uncompensated and apply compensation later. Built-in compensation is proprietary software that cannot be validated, and so it is difficult to tell whether compensation is performed correctly; moreover, in those cases where it goes wrong, it is not possible to re-compensate correctly without the original data. Similarly, at present most commercial flow cytometry software does not compensate for autofluorescence, so such compensation mechanisms should not be used in the presence of non-trivial autofluorescence.

#### 4.2.2 Conversion to absolute units

The measurements returned by a flow cytometer are highly relative and subject to change: not only do they depend on the machine, its configuration, and the laser and detector settings for a particular assay, but also the instruments tend to drift in calibration over time as well. Various standard fluorescent beads have been developed to deal with this calibration problem, and have been demonstrated to provide standardizable precise measurements across a wide range of instruments and channels (Schwartz et al., 2004; Vogt et al., 2008; Wang et al., 2008; Hoffman et al., 2012).

Critically, the bead manufacturer SpheroTech provides certain classes of beads (e.g., RCP-30-5A) that have been calibrated to equivalent molecules of various standard fluorescent stains (SpheroTech, 2001). These bead samples contain a mixture of beads with multiple distinct levels of fluorescence and non-uniform gaps between levels. This means that a linear conversion from relative units to absolute units, such as Molecules of Equivalent Fluorescein (MEFL) can be computed simply by finding the peaks in the appropriate channel of a fluorescence histogram (e.g., the FITC channel for MEFL) and matching against the list of calibration levels. Figure 7C shows such an example of peak identification on a sample of SpheroTech RCP-30-5A beads. Note the uneven gaps between peaks, which allow unique identification even when only a few peaks are visible.

Standard fluorophore measurements, however, do not provide comparable units between channels. Rather, each channel is characterized with respect to a different fluorescent stain and the relationship between fluorescent stains is in general different from the relationship between the various fluorescent proteins. Further, the fluorescence of various fluorescent proteins may depend on the context in which they are expressed.

The TASBE method obtains equivalent units by selecting one of the standard units (MEFL is recommended, as its greenish/yellow range is one of the most widely used channels) and computing a linear conversion factor from other channels via a multi-color control. A multi-color control must strongly constitutively express both the fluorescent protein measured in the standard channel and at least one other fluorescent protein. Each fluorescent protein in the multi-color control must be expressed using equivalent promoters and context. In some contexts, such as mammalian cells, this is relatively easy: there is little interaction between promoter and coding sequence in an expression cassette, and each expression cassette can be placed in its own plasmid and cotransfected. In bacterial cells, on the other hand, where the interaction with the 5′UTR is more significant and cotransfection typically is extremely difficult, it is first necessary to validate that there is sufficient insulation between the fluorescent proteins by comparing different constructs. Figure 7D shows an example of finding a linear conversion factor from compensated Pacific Blue arbitrary units to compensated FITC arbitrary units in mammalian HEK293 cells, using a cotransfection of two plasmids, one expressing EBFP2, the other EFYP, both under the same strong promoter. Multiplying by the conversion factor changes Pacific Blue arbitrary units to FITC arbitrary units, which can then be converted to MEFL and thus allowing blue and yellow fluorescent proteins to be measured in equivalent absolute units.

Finally, note that it is certainly possible to go beyond measurements of equivalent fluorescence to estimates of number of molecules. In many cases, this may be desirable to do (e.g., Rosenfeld et al., 2007), but it is not always necessary and may introduce additional noise. It is not always necessary because the base requirement for effective modeling is absolute units, and MEFL is already such. Further, fluorescence is being measured directly, while molecule counts are inferred based on additional estimates; differences in chemical environment, quenching, and other such factors may affect the fluorescence per molecule, however, and can create distortions in molecule estimates.

Putting it all together, these four stages of the TASBE method, applied following the workflow in Figure 6, provide absolute unit measurements from large numbers of single cells. This method thereby provides an example of a measurement assay sufficient to serve as a foundation for the development of model-driven engineering methods, as will be shown in the next section.

## 5 From Measurement to Prediction and Device Engineering

The results of calibrated large-scale per-cell measurement assays can provide a firm foundation for the development of model-driven engineering methods. This section demonstrates this relationship though presentation of three recent examples of ways in which model-driven engineering methods have been derived from calibrated flow cytometry data: improving the quality of data obtained from assays through intra-sample validation, predicting multi-component systems from models of individual components, and debugging of complex novel components.

### 5.1 Intra-sample validation

One of the frequent frustrations in biological experiments is the difficulty in distinguishing between effects due to the intended subject of study versus those due to fluctuations and errors in protocol or reagents. This difficulty can happen at any scale, from individual samples to correlated sets of samples, to entire replicates or experiments. Standard controls can help to identify problems, but cannot detect problems that do not affect the control or affect it more subtlely.

With the capability to measure and compare absolute fluorescent distributions across experiments, however, it is possible to validate each individual sample using the distribution of fluorescence within the sample. This can be implemented, for any assay not expected to have a strong impact on cell viability, by including a strong constitutively expressed fluorescent protein in the test construct. Often, this can even be done without modifying the system under study at all, because such a fluorescent protein is already included as a transfection marker.

Once a baseline model of variation for fluorescent distributions has been established (e.g., from single-positive controls of the constitutive protein), then each sample can be validated individually by evaluating its distribution of constitutive fluorescence. Whenever there is a significant difference from baseline, it indicates either that something has varied significantly in the protocol or that there is a strong impact on cell viability (e.g., resource competition, disabling of function in expression machinery). Not only can this method detect problems with individual samples but can also detect problems that may not be visible from population-averaged data alone. For example, contamination or sample degradation may not appear to have a significant effect on the mean, but may contain anomalous “bumps” in other portions of the distribution.

**Figure 8:**
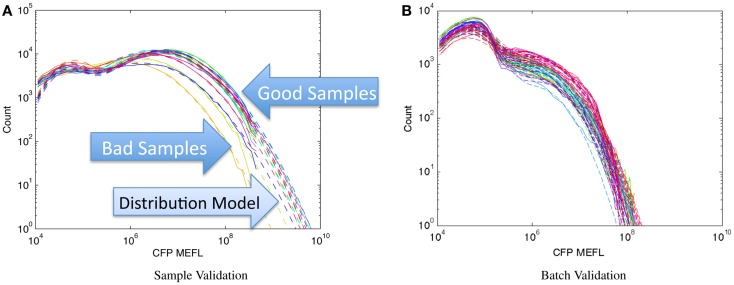
**Population distribution of a constitutive fluorescent protein (CFP) can be used to identify protocol problems, from individual samples (A) to entire replicates (B)**. Data shown are from sample material on Adler et al. (2014): solid lines are observed distribution, dashed lines are bimodal model fit, used for quantitative comparison of sample to expected distribution.

Figure 8 shows examples of applying this method to assays of mammalian cells that have been cotransfected with both a circuit under study and a constitutive transfection marker, showing the expression histogram of the constitutive marker for each sample in the experiment in a unique color. In each case, all of the samples in an experiment are compared on a single graph and fitted against a bimodal log-normal distribution model of the expected transfection distribution, where the upper component is successfully transfected cells and the lower component is untransfected cells. For the experiments shown, the baseline model has been established as the active component containing at least 50% of the cells and having a geometric mean of approximately 10^7^ MEFL. Figure 8A shows an experiment with mostly normal transfections and a small number of anomalous samples: the three lowest samples, whose distributions are clearly different than the rest, are rejected while the rest are retained. Figure 8B shows a much more extreme problem: an entire batch of data with a significantly degraded transfection efficiency.

This approach thus constitutes a model-driven engineering method, though not one directly tied to design. Rather, being able to compare assays of per-cell sample data against an absolute distribution model enables both more principled sample rejection and early detection of problems that might otherwise lead to large amounts of wasted time and effort.

### 5.2 Prediction of multi-component systems

Distribution models can also, of course, be applied directly to system design, by using them to predict the consequences of different design choices. By allowing examination of the different modes of variation in the distribution of expression in a population of cells, calibrated flow cytometry also allows more parameters relevant to the mechanisms regulating expression to be quantified.

**Figure 9:**
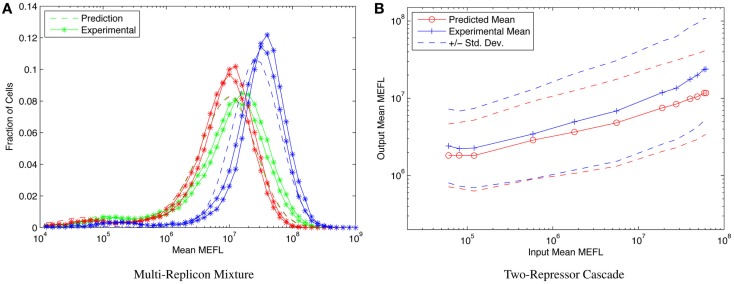
**Component models built using calibrated flow cytometry data enable high-precision predictions of multi-component systems**. For example, **(A)** models of single- and dual-replicon transfection can predict (dashed lines) the observed histogram for distribution of fluorescence (lines with stars, two replicates) in a three-replicon mixture (figure from Beal et al. (2014), and **(B)** repressor models can predict the observed mean and population distribution of fluorescent expression of combinational circuits such as the two-repressor cascade shown [figure from Davidsohn et al. (2014)].

Consider, for example, the highly asymmetric expression distribution shown in Figure 5B, from Beal et al. (2014). The asymmetries in the distribution correspond to separable effects of different mechanisms regulating expression from Sindbis replicons transfected into BHK-21 mammalian cells, as described in Section 1. Combining such cotransfection data with time-series observations of single replicon expression suffices to construct a model for the expression over time of any dosage mixture of constitutive replicons. This model, in turn, allows high-precision prediction and engineering of the distribution of fluorescent expression, evolving over time, across a wide range of multi-replicon mixtures. An example of such prediction is shown in Figure 9A, which compares the predicted and experimentally observed distributions of fluorescent expression from a mixture of 360 ng of mVenus replicon (green), 360 ng of mKate replicon (red), and 1080 ng of EBFP2 replicon (blue), observed 50 h after transfection into BHK-21 cells.

Another example is prediction of cotransfected circuits in HEK293 mammalian cells from characterization of individual repressors, as presented in Davidsohn et al. (2014). Here, calibrated flow cytometry was applied to produce a mixed mechanistic/phenotypic model of the action of three-repressor/promoter pairs (actually synthetic hybrid activator/repressor systems with promoters activated by constitutive VP16Gal4 and repressed by one of the transcriptional regulators TAL14, TAL21, or LmrA). The distribution model is parametrized by time, by input expression as indicated by a co-expressed input fluorescent protein, and by relative circuit dosage as indicated by a constitutive fluorescent protein. Models of individual repressors may then be combined to create a model of a multi-repressor circuit, allowing high-precision prediction of both the population mean and the cell-to-cell variation of output protein expression for combinational circuits, as demonstrated in Davidsohn et al. (2014) for multiple two-repressor cascades and three-repressor feed-forward circuits, such as the TAL21–TAL14 cascade shown in Figure 9B.

From such predictions comes the ability to eliminate configurations that are not productive to assay, and to prioritize assays for those constructs mostly likely to prove successful, as discussed in Section 2, providing another example of model-driven engineering based on calibrated flow cytometry.

### 5.3 Improving device engineering

The deeper insight enabled by distribution models can also support model-driven engineering by helping to engineer devices with less mutual constraint, which decreases the expected difficulty of discovering acceptable system configurations as discussed in Section 2. Here, the value of being able to compare population distributions is that different aspects of the behavior of a regulatory device can affect the structure of the distribution in different ways. For example, when a constitutive fluorescent protein is included in a high-variance cotransfection, it enables the behavior of the device to be examined as a function of the relative number of circuit copies. Since different aspects of a device’s behavior scale differently with copy numbers, such sub-sample decomposition can provide deeper insight into the causes of observed behavior. For example, the degree of leakage in a repressor can be quantified by the location of the inflection point where expression rises above autofluorescence in a “minus” sample. Such forms of analysis, in turn, allow the relative importance of different performance limitations to be evaluated, enabling better focusing of engineering efforts on the limiting factors in the design of a device.

For example, Kiani et al. (2014) presents new classes of repressor devices based on the CRISPR system, which are characterized using calibrated flow cytometry. Each device comprises a number of different components, including constitutive expression of the mutant protein Cas9m, which forms a targeted repressor when it binds with gRNA expressed either directly or from introns, Gal4VP16, which drives device expression unless overridden by Cas9m, and a complex hybrid promoter including multiple targeting sites for both Cas9m and and Gal4VP16. In the early stages of designing these new devices, there was a problem with high variability with respect to gRNA sequence: some gRNAs performed very well, others inexplicably poorly. An assay with a constitutive marker revealed that the less functional devices had a much lower rate of leakage expression at the input stage, indicating that the problem lay not in the action of the repressor complex on the promoter, where it was originally believed the problem lay, but in the expression of intronic gRNA. Refocusing engineering effort on that aspect of the system led to new versions of devices with greatly improved performance, which are the ones ultimately reported in Kiani et al. (2014).

Thus, just as in the previous examples, absolute unit comparison of distributions of cell behaviors enables a more model-driven approach to engineering biological organisms. The only difference is that in this case, the set of interacting mechanisms that the methods are applied to is being conceived of by the engineers as a single complex “device.”

## 6 Discussion and Directions for Future Development

As the field of synthetic biology continues to expand, the problems of measurement and design are becoming increasingly pressing. This paper has developed an information-based measure that can be used to determine the relative importance of good design methods in synthetic biology applications. Applying this measure shows that precision modeling and design must play an important role in future application development. Progress in modeling and design has previously been inhibited by limitations in assay protocols that made it difficult to effectively study the distribution of expression levels within a cell population. Calibrated flow cytometry, however, is an example of a recently developed method that overcomes this limitation, and applications of this method demonstrate how comparison of expression level distributions can enable deeper insight into cell behavior as well as high-precision modeling and design.

### 6.1 Directions for future development

The future of synthetic biology engineering rests on three complementary pillars of development: high-throughput screening, improved device families, and precision modeling and design. The first two of these are already the subjects of heavy investigation and rapid progress, while the third has proved more elusive. Recent results from calibrated flow cytometry, however, indicate that there is now a sufficient foundation for renewed investigation of precision modeling and design.

Strategic investment in this area has the potential for transformative impact across a broad space of applications for engineered biological organisms. With respect to calibrated flow cytometry in particular, there are three key directions for work:

- Exploitation and integration of calibrated flow cytometry: calibrated flow cytometry is a readily accessible technology, as it builds on instruments and methods already widely in use, requiring only a few simple additional controls. When combined with more sophisticated data analysis, it has the potential to radically improve the amount of insight and precision of models that can be derived from experiments, as illustrated in Section 5. Significant impact is thus likely to be obtained from the dissemination and exploitation of calibrated flow cytometry techniques, and their integration with a wide variety of systems and synthetic biology projects.
- Application to a broader range of organisms: at present, calibrated flow cytometry has been applied primarily to the engineering of sensing and control circuits in mammalian cells. Preliminary work has already begun on extension to other cell types, as discussed in Section 4, each of which may require its own modifications and refinements in order to operate correctly: for example, multi-color controls are harder to calibrate in bacteria, while plant cells have extremely strong autofluorescence due to chlorophyll.
- Application beyond sensing and control circuits: calibrated flow cytometry should also be applicable to problems outside of the realm of sensing and control circuits, though it will likely need to be used in combination with other assays. For example, it should be possible to examine population distributions for chemical synthesis with calibrated metabolite sensors, or to apply calibrated flow to tissue engineering by using it in combination with imaging.

Calibrated flow cytometry, of course, is just one of many potential assays for obtaining information to enable high-precision modeling and design. In fact, fluorescence is far from an ideal quantity for such assays, as it is often only a proxy measure for other, more relevant properties of cells. An important longer term goal is thus to develop new assays that can provide the same power to examine population distributions, but for other quantities such as molecule count or the configuration of sub-cellular structures.

## Conflict of Interest Statement

The author declares that the research was conducted in the absence of any commercial or financial relationships that could be construed as a potential conflict of interest.

## Footnotes

^1^The assumption of random exploration may at first seem strange to those unfamiliar with information theory, since any engineering effort is of course guided by knowledge, experience, and educated guesses. In this information-based formulation of the engineering problems, however, all such guidance is encoded by the weight function. Whatever, choice remains is arbitrary and cannot in general outperform random choice.

^2^For example, most tools for designing computational circuits have focused on digital logic, rather than analog or hybrid systems.

^3^The RFU method (Kelly et al., 2009) attempts to avoid this necessity by comparing with a standard constitutive promoter; the behavior of that promoter, however, may change radically from context to context.
